# Effect of *RAGE* gene polymorphisms and circulating sRAGE levels on susceptibility to gastric cancer: a case–control study

**DOI:** 10.1186/s12935-017-0391-0

**Published:** 2017-02-06

**Authors:** Taijie Li, Weijuan Qin, Yanqiong Liu, Shan Li, Xue Qin, Zhiming Liu

**Affiliations:** 1 Department of Clinical Laboratory, The First Affiliated Hospital of Guangxi Medical University, Nanning, Guangxi China; 2 Department of General Surgery, The First Affiliated Hospital of Guangxi Medical University, Nanning, Guangxi China

**Keywords:** RAGE, Polymorphism, sRAGE, Levels, Gastric cancer

## Abstract

**Background:**

To investigate the influence of polymorphisms in the receptor for advanced glycation end products (RAGE) gene and circulating soluble RAGE (sRAGE) levels on susceptibility to gastric cancer, and identify whether these polymorphisms were correlated with serum sRAGE levels.

**Methods:**

We performed a hospital-based case–control study involving 200 gastric cancer patients and 207 cancer-free controls. Four well-characterized *RAGE* genetic polymorphisms, namely, rs1800624, rs1800625, rs184003, and rs2070600 were genotyped by PCR–RFLP.

**Results:**

The rs2070600 AG genotype might play a predominant role in the development of gastric cancer (adjusted OR 1.62, 95% CI 1.03–2.58). In contrast, the rs184003 GT genotype represented significantly reduced risk for gastric cancer (adjusted OR 0.62, 95% CI 0.39–0.99). Subgroup analysis demonstrated that rs2070600 AG variant genotype enhanced the gastric cancer risk among nonsmokers (OR 1.71, 95% CI 1.01–2.91), nondrinkers (OR 1.75, 95% CI 1.03–2.97), and patients with tumor stage III (OR 2.00, 95% CI 1.13–3.56). The average sRAGE levels in the gastric cancer patients were significantly decreased compared with those of the healthy controls. Subjects carrying the rs2070600 AG genotype had a decreased ability to produce sRAGE. Subjects carrying the rs184003 T variant allele had an increased ability to sRAGE.

**Conclusions:**

These findings suggested that the variant genotypes of rs184003 and rs2070600 in the *RAGE* gene exhibit significant associations with gastric cancer risk and circulating sRAGE levels inverse change simultaneously, leading to a marked causal estimate between lowered sRAGE levels and increased gastric cancer risk.

**Electronic supplementary material:**

The online version of this article (doi:10.1186/s12935-017-0391-0) contains supplementary material, which is available to authorized users.

## Background

Gastric cancer is a major cause of cancer deaths worldwide, with almost one million new cases estimated to have occurred worldwide in 2012, making it the fifth most common malignancy [1]. More than 70% of cases (677,000 cases) occur in developing countries (456,000 in men and 221,000 in women), and half the world total occurs in Eastern Asia (mainly in China) [1]. The prevalence of the disease highlights the importance of a better understanding of the risk factors related to gastric cancer development.

Gastric carcinogenesis is a multifactorial, complex, multistep event involving genetic and environmental factors [2]. The receptor for advanced glycation end-products (RAGE) is a member of the immunoglobulin superfamily of cell surface receptors, and its interaction with advanced glycation end products and other molecules plays a role in the pathogenesis of tumorigenesis and metastasis [3–5]. Numerous clinical studies have established a strong association between RAGE expression and the malignant potential of various cancer types, such as gastric cancer [5], prostate cancer [6], gallbladder cancer [7], pancreatic cancer [8], non small cell lung cancer [9], and colorectal cancer [10]. In humans, the gene coding for RAGE, which has been shown to be highly polymorphic, is located on chromosome 6p21.3 in the major histocompatibility complex locus class II/III junction [11]. Research suggests that polymorphisms within ligand-binding regions of the *RAGE* gene may affect the expression and function of RAGE [12]. Thus, *RAGE* polymorphisms may represent potential candidates as causes of various types of cancers. In this regard, several epidemiological studies have investigated the association between the *RAGE* gene polymorphism and the risk of various cancers, such as hepatocellular carcinoma [13], breast cancer [14], oral cancer [15], colorectal cancer [16], ovarian cancer [17], and lung cancer [18]. Other studies that examined the genetic background of the *RAGE* gene found that the circulating soluble form of RAGE (sRAGE) was largely determined by *RAGE* genetic defects [19–21].

Thus far, only one study has investigated the association between *RAGE* polymorphisms and gastric cancer risk. In 2008, Gu et al. reported that individuals with the rs2070600 variant genotypes (82Gly/Ser and 82Ser/Ser) had a significantly higher risk of gastric cancer (adjusted odds ratio [OR] 1.47, 95% confidence interval [CI] 1.05–2.06) [22]. However, this study evaluated only one single nucleotide polymorphism, (SNP) in the *RAGE* gene in a northeastern Chinese population.

The aims of the present case–control study were to determine (1) the relationship between four well-characterized polymorphisms in the *RAGE* gene (rs1800624 [−374T>A], rs1800625 [−429T>C], rs184003 [1704G>T], and rs2070600 [Gly82Ser]) and the risk of gastric cancer in a southwest Han Chinese population; (2) the association between sRAGE levels and gastric cancer; and (3) the association between *RAGE* polymorphisms and sRAGE levels.

## Methods

### Study participants

This was a hospital-based case–control study. The cases were inpatients newly diagnosed with histologically confirmed gastric cancer, consecutively recruited from the First Affiliated Hospital of Guangxi Medical University, Guangxi, China between April 2015 and December 2015. Patients were excluded if they had any of the following: (1) concomitant malignant neoplasias, (2) acquired immunodeficiency syndrome, and (3) acute or chronic inflammatory diseases. Controls were randomly recruited from a pool of healthy volunteers without clinical evidence of any cancer who visited the general health check-up centers at the same hospital during the same period of the study all the participators provided written informed consent. This case–control study was approved by the ethics committee of The First Affiliated Hospital of Guangxi Medical University.

For each participant, demographic features, laboratory data, and pathological findings were obtained from electronic medical records. The demographic data included age, gender, ethnicity, body mass index (BMI), family history of cancer, smoking status, and drinking status. The laboratory data included information on carcinoembryonic antigen and carbohydrate antigen 199 levels. The pathological findings recorded were the tumor location (upper, middle, lower, and whole), tumor node metastasis stage, and differentiation.

### Selection of tag SNPs selection

SNP genotype information was retrieved from the dbSNP database (http://www.ncbi.nlm.nih.gov/projects/SNP/) and HapMap database (http://hapmap.ncbi.nlm.nih.gov/). The selection of the SNPs was based on them being well-characterized, well-known, and common functional SNPs, as well as on an extensive literature study, their population frequency based on a minor allele frequency of >5%, and a previously described association with cancer. Based on the aforementioned criteria, four SNPs were selected: rs2070600 (Gly82Ser) in the third exon, rs184003 (G1704A) in the seventh intron, rs1800624 (T-374A) in the promoter region, and rs1800625 (T-429C) in the promoter region.

### Determination of sample size

The sample size was estimated using Quanto software (version 1.2.4) based on a probability of α = 0.05 and β = 0.10. The case–control design used an approximately 1:1 ratio. The assumed prevalence of the SNP rs2070600GG genotype in the control group was 0.488 (HapMap Project dbSNP database: http://www.ncbi.nlm.nih.gov/SNP/). The inheritance model was recessive. The estimated marginal genetic effect was 2.0. The type I error rate was 0.05 (two-sided). According to the above parameters, the power analysis showed that a sample size of 134 would have more than an 80% power to detect a genotype-related risk.

### Sample collection

In total, 5 ml of blood (5 ml) were drawn into ethylene diaminetetraacetic acid-coated vials from the patients and control subjects. Two milliliters of the whole-blood samples were stored at −20 °C until DNA isolation, and 3 ml of each sample were placed in drying tubes and centrifuged for 10 min at 3000*g*. The serum was extracted and stored at −20 °C for sRAGE analysis using an enzyme-linked immunosorbent assay (ELISA).

### DNA isolation and *RAGE* genotyping

DNA was isolated from peripheral leukocytes using the phenol–chloroform protocol, as described in our previous study [23]. The concentration and purity of the DNA were determined spectrophotometrically. The obtained DNA was stored at −20 °C until it was analyzed using the polymerase chain reaction (PCR). The *RAGE* rs2070600, rs184003, rs1800624, and rs1800625 polymorphisms were screened using a thermocycler PCR system, followed by a restriction fragment length polymorphism (RFLP) assay. For each SNP, the PCR was conducted in a reaction volume of 25 μl, consisting of 1 μl of each specific primer, 2 μl of genomic DNA, 12.5 μl of Green PCR Master Mix (Shanghai Sangon Biotech Co., Ltd., China), and 8.5 μl of nuclease-free water. The specific primers for rs2070600 were: forward, 5′-GAAGGTCCTGTCTCCCCAG-3′; reverse, 5′-GTAAGAGGGAGGCCTTGGAG-3′. For the rs184003, the primers used were: forward, 5′-GAGACAGGGCTCTTCACACT-3′; reverse, 5′-TTTCCCTCGTTAGCCCTCTG-3′. For the rs1800624 and rs1800625, the primers used were: forward, 5′-GGGCAGTTCTCTCCTCACTT-3′; reverse, 5′-CGTCTTGTCACAGGGAATGC-3′. The PCR conditions for these four SNPs were all as follows: initial denaturation at 95 °C for 5 min, 30 cycles of denaturation at 95 °C for 30 s, annealing at 61 °C for 30 s, extension at 72 °C for 45 s, and a final extension at 72 °C for 10 min.

Restriction enzymatic digestion of all the PCR products was performed using 1 μl of restriction enzymes (Thermo Scientific): AluI for rs2070600 and rs1800625, *FspB*I for rs184003, and *Mun*I for rs1800624.The digested fragments were directly separated by electrophoresis in 3% agarose gel and visualized in ultraviolet light after GoldView I staining. For rs2070600, the sizes of the fragments were 149 + 63 bp for the G allele and 212 bp for the A allele. For rs184003, the sizes of the fragments were 35 + 467 bp for the G allele and 502 bp for the T allele. For rs1800624, the sizes of the fragments were 212 + 289 bp for the T allele and 501 bp for the A allele. For rs1800625, the sizes of the fragments were 342 + 159 bp for the C allele and 501 bp for the T allele.

### DNA sequencing

To determine the accuracy of the PCR–RFLP method, about 10% of the samples were randomly selected, and the genotypes were verified by the direct sequencing method with an ABI Prism 3100 (Applied Biosystems, Shanghai Sangon Biological Engineering Technology & Services Co., Ltd., China). The resultant genotypes showed no differences.

### Measurement of serum sRAGE levels

The sRAGE levels in 90 sera samples from the gastric cancer patients and 90 sera samples from the controls were measured by a double-sandwich ELISA (Cusabio Biotech Co., Ltd., Wuhan, China), following the manufacturer’s instructions and using a standard range of 0.0–5000 pg/ml. The readers of the laboratory assay were blinded to the clinical data.

### Statistical analysis

Continuous variables were expressed as means ± standard deviations (SD) or medians ± interquartile ranges. Group differences and normally distributed data were analyzed by the Student’s *T* test, and the Mann–Whitney *U* test was applied for data not normally distributed. Categorical variables were presented as frequencies and were compared between patients and controls by the χ^2^ test and Fisher’s exact test, when appropriate. The χ^2^ test was used to test the Hardy–Weinberg equilibrium at each locus using a contingency table of observed-versus-expected genotypic frequencies.

The genotypes of the four examined SNPs in the *RAGE* gene were explored by binary logistical regression analyses to obtain the ORs and their 95% CIs under the assumptions of codominant, dominant, and recessive models of inheritance after adjusting for age, gender, BMI, ethnicity, family history of cancer, smoking status, and drinking status. To evaluate the joint effects of the four SNPs in the *RAGE* gene, SHEsis software (http://analysis.bio-x.cn/myAnalysis.php) [24] was employed to construct haplotypes between the patients and controls. To avoid chance findings, only common haplotypes with frequencies ≥0.03 in all the study participants were analyzed. To investigate the effect of other potential confounding variables on the association between the RAGE polymorphisms and gastric cancer risk, the population was stratified according to gender, smoking status, and drinking status. SPSS version 16.0 for Windows (SPSS Inc., IL, USA) software was used for all the statistical analyses. A two-sided P value of <0.05 was accepted as statistically significant.

## Results

### Characteristics of the study participants

Table 1 shows the demographic and clinical characteristics of all the subjects in the study. In total, 200 gastric cancer patients and 207 controls were enrolled in the study. There were no differences between the two groups in terms of mean age, smoking status, drinking status, and ethnicity. However, there was a higher proportion of males and a family history of cancer among the patients with gastric cancer than among the controls. The patient groups had a significantly lower BMI and higher serum levels of carcinoembryonic antigen and carbohydrate antigen 199 when compared to those of the healthy controls. Among the 200 gastric cancer cases, 20 (0.010), 145 (0.725), 30 (0.150), and 5 (0.025) patients had undifferentiated, well-differentiated, moderately differentiated, and poorly differentiated carcinomas, respectively; 26 (0.130), 35 (0.175), 85 (0.425), and 54 (0.270) patients had stage I, II, III, and IV tumors, respectively.

**Table 1:** Baseline characteristics of the study population

Characteristics	Gastric cancer	Controls	P value
Total number	200	207	
Age (mean ± SD, years)^a^	54.43 ± 11.77	53.23 ± 4.335	0.170
BMI (mean ± SD, kg/m^2^)^a^	20.49 ± 3.11	22.37 ± 3.44	<0.001
Gender			
Male	131 (0.655)	97 (0.469)	<0.001
Female	69 (0.345)	110 (0.531)	
Smoking status			
Yes	59 (0.295)	64 (0.309)	0.755
No	141 (0.705)	143 (0.691)	
Drinking status			
Yes	52 (0.260)	58 (0.280)	0.647
No	148 (0.740)	149 (0.720)	
Ethnicity			
Han	97 (0.485)	104 (0.502)	0.940
Zhuang	91 (0.455)	91 (0.440)	
Other	12 (0.060)	12 (0.058)	
CEA (median ± IQR, ng/ml)^b^	2.79 ± 3.53	2.20 ± 2.71	<0.001
CA199 (median ± IQR, ng/ml)^b^	9.94 ± 17.91	3.17 ± 7.64	<0.001
Family history of cancer			
Yes	14 (0.070)	4 (0.019)	0.013
No	186 (0.930)	203 (0.981)	
Tumor location			
Upper	185 (0.925)		
Middle	6 (0.030)		
Lower	9 (0.045)		
Differentiation			
Undifferentiated	20 (0.010)		
Poor	145 (0.725)		
Moderate	30 (0.150)		
Well	5 (0.025)		
Clinical state			
I	26 (0.130)		
II	35 (0.175)		
III	85 (0.425)		
IV	54 (0.270)		

*SD* standard deviation, *IQR* interquartile range, *NA* not available

^a^Student’s *t*-test

^b^Mann–Whitney *U* test

### *RAGE* polymorphisms and gastric cancer risk

The observed genotype distributions of the SNPs rs2070600, rs184003, rs1800624, and rs1800625 in the *RAGE* gene were consistent with the Hardy–Weinberg equilibrium in the controls (P > 0.05). The genotype and allele frequencies of the four polymorphisms in the patients and controls are shown in Table 2. In the logistic regression analyses, a moderately higher risk of gastric cancer was observed in the AG carriers of rs2070600, with GG used as a reference (OR 1.62, 95% CI 1.03–2.58; P = 0.038), after adjustment for gender, age, BMI, family history of cancer, ethnicity, smoking status, and drinking status. Moreover, subjects carrying at least one copy of the A allele for the rs2070600 SNP (dominant model: AG + AA vs. GG) were 1.56 times more likely to develop gastric cancer (OR 1.56, 95% CI 1.01–2.39, P = 0.044). In contrast, after adjustment for the above-mentioned variables, the rs184003 GT genotype was associated with a significantly reduced risk of gastric cancer (OR 0.62, 95% CI 0.39–0.99; P = 0.048). However, there were no significant differences in the genotype and allele distributions of the other two polymorphisms (rs1800624 and rs1800625) between the cases and controls (P > 0.05).

**Table 2:** Genotype distributions and allele frequencies of *RAGE* polymorphisms between cases and controls

Model	Controls (N = 207)	Cancer (N = 200)	Adjusted OR (95% CI)^a^	P
rs2070600				
GG	136 (0.657)	113 (0.565)	1.00^ref^	
AG	58 (0.280)	72 (0.360)	1.62 (1.03–2.58)	*0.038*
AA	13 (0.063)	15 (0.075)	1.27 (0.54–2.98)	0.581
A allele	84 (0.203)	102 (0.255)	1.00^ref^	
G allele	330 (0.797)	298 (0.745)	1.37 (0.96–1.95)	0.082
AG + AA vs. GG			1.56 (1.01–2.39)	*0.044*
AA vs. AG + GG			1.09 (0.47–2.51)	0.841
rs184003				
GG	138 (0.667)	148 (0.740)	1.00^ref^	
GT	64 (0.309)	48 (0.240)	0.62 (0.39–0.99)	*0.048*
TT	5 (0.024)	4 (0.020)	1.02 (0.24–4.27)	0.984
G allele	340 (0.821)	344 (0.860)	1.00^ref^	
T allele	74 (0.179)	56 (0.140)	0.72 (0.48–1.08)	0.107
GT + TT vs. GG			0.64 (0.41–1.02)	0.060
TT vs. GT + GG			1.15 (0.28–4.81)	0.849
rs1800624				
TT	166 (0.802)	150 (0.750)	1.00^ref^	
AT	35 (0.169)	42 (0.210)	1.40 (0.83–2.39)	0.210
AA	6 (0.029)	8 (0.040)	1.03 (0.32–3.35)	0.961
T allele	367 (0.886)	342 (0.855)	1.00^ref^	
A allele	47 (0.114)	58 (0.145)	1.25 (0.81–1.94)	0.319
AA + AT vs. TT			1.34 (0.82–2.21)	0.246
AA vs. AT + TT			0.97 (0.30–3.12)	0.952
rs1800625				
CC	1 (0.005)	3 (0.015)	1.00^ref^	
CT	22 (0.106)	13 (0.065)	0.25 (0.02–2.97)	0.275
TT	184 (0.889)	184 (0.920)	0.44 (0.04–4.70)	0.500
C allele	24 (0.058)	19 (0.048)	1.00^ref^	
T allele	390 (0.942)	381 (0.953)	1.33 (0.69–2.54)	0.393
TT + CT vs. CC			0.42 (0.04–4.45)	0.473
TT vs. CT + CC			1.52 (0.75–3.08)	0.245

Italic values indicate a significant difference

^a^Adjusted for gender, age, BMI, family history of cancer, ethnicity, smoking and drinking status

### Stratified analyses

To investigate the effect of other potential confounders on the association between the *RAGE* polymorphisms and gastric cancer risk, the population was stratified according to gender, smoking status, and drinking status. In addition, to better understand the prognosis of gastric cancer, the relationship between the *RAGE* genotype polymorphisms and cancer stage was assessed. Additional files 1, 2 and 3 present the results of the subgroup analysis by smoking status, drinking status, and gender. Nonsmokers with the rs2070600 AG genotype but not smokers with this genotype showed a significantly elevated risk of gastric cancer (adjusted OR 1.71, 95% CI 1.01–2.91; P = 0.043). As regards drinking status, in nondrinkers, the presence of the rs2070600 variant genotypes was associated with a significantly increased risk of gastric cancer (AG vs. GG: OR 1.75, 95% CI 1.03–2.97; dominant model AG + AA vs. GG: OR 1.66, 95% CI 1.01–2.74), whereas the association was not statistically significant among drinkers. No effect of gender on the association between the *RAGE* polymorphism and susceptibility to gastric cancer was observed. Additional file 4 presents the results of the stratified analyses by the cancer stage. As shown by the binary analysis, AG carriers of rs2070600 had a markedly higher risk of stage III gastric cancer (OR 2.00 95% CI 1.13–3.56; P = 0.018), using GG as a reference, after adjustment for age, gender, BMI, family history of cancer, ethnicity, drinking status, and smoking status. The gene polymorphisms of rs184003, rs1800624, and rs1800625 did not affect the risk of gastric cancer at different stages.

### *RAGE* haplotypes and gastric cancer risk

Haplotypes of the four polymorphisms in the *RAGE* gene were derived to detect haplotypes specifically correlated with gastric cancer. Their frequencies (≥3%) are summarized in Table 3. Four haplotypes were identified in the order rs2070600, rs184003, rs1800624, and rs1800625, with the GGTT haplotype the most prevalent in both gastric cancer patients and controls. However, no statistically significant association was found between the haplotypes and gastric cancer risk.

**Table 3:** Analysis of *RAGE* haplotype frequencies with the risk of gastric cancer

Haplotype	Case (frequency)	Control (frequency)	Chi^2^	Pearson’s p	OR (95% CI)
AGTT	89 (0.222)	79 (0.190)	1.714	0.190	1.26 (0.89–1.77)
GGAT	52 (0.128)	44 (0.106)	1.220	0.269	1.27 (0.83–1.96)
GGTC	15 (0.038)	23 (0.056)	1.141	0.285	0.70 (0.36–1.35)
GGTT	180 (0.449)	194 (0.469)	0.057	0.811	0.97 (0.73–1.28)
GTTT	50 (0.125)	69 (0.166)	2.336	0.126	0.74 (0.50–1.09)

### Association between sRAGE levels and gastric cancer

**Fig. 1:**
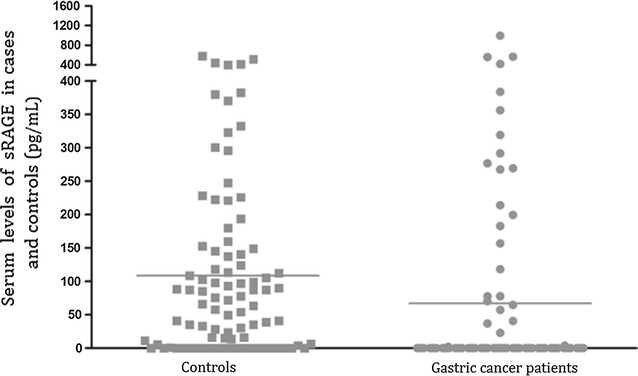
sRAGE levels in health controls and gastric cancer patients (pg/ml)

The average sRAGE levels in the gastric cancer patients were 56.86 ± 147.74 pg/ml, and they were 108.31 ± 132.97 pg/ml in the controls (Table 4; Fig. 1). The serum sRAGE levels in the controls were significantly higher than those in the gastric cancer patients (P = 0.015).

**Table 4:** The association between RAGE gene polymorphisms and sRAGE levels

Polymorphisms	Controls (N = 90)		Cancer (N = 90)	
	sRAGE levels (pg/ml)	P value	sRAGE levels (pg/ml)	P value
sRAGE levels (mean ± SD)	108.31 ± 132.97		56.86 ± 147.74	*0.015*
rs2070600				
GG	133.72 ± 137.73	1.00^ref^	90.56 ± 183.54	1.00^ref^
AG	49.13 ± 104.88	*0.010*	10.96 ± 39.47	*0.024*
AA	27.22 ± 30.56	0.130	0.00 ± 0.00	0.171
rs184003				
GG	89.68 ± 114.24	1.00^ref^	32.46 ± 77.92	1.00^ref^
GT	145.13 ± 160.93	0.071	119.50 ± 254.34	*0.016*
TT	379.64 ± 0.00	*0.014*	124.63 ± 200.63	*0.044*
rs1800624				
TT	98.32 ± 132.76	1.00^ref^	45.23 ± 117.97	1.00^ref^
AT	171.68 ± 138.67	0.072	49.72 ± 92.55	0.872
AA	84.75 ± 63.20	0.840	203.13 ± 393.90	0.022
rs1800625				
CC	105.09 ± 0.00	1.00^ref^	0.00 ± 0.00	1.00^ref^
CT	34.82 ± 34.43	0.089	5.29 ± 13.99	0.545
TT	116.62 ± 138.25	0.934	63.50 ± 155.47	0.484

Italic values indicate a significant difference

### Association between *RAGE* polymorphisms and sRAGE levels

As shown in Table 4, decreased levels of sRAGE were found in the gastric cancer subjects carrying the rs2070600 AG genotype (49.13 ± 104.88 pg/ml) compared with those carrying the AA genotype (133.72 ± 137.73 pg/ml; P = 0.01). In addition, the serum sRAGE level of the gastric cancer patients with the rs184003 TT genotype was significantly higher (379.64 ± 0.00 pg/ml) than that of the subjects with the wild-type GG genotype (89.68 ± 114.24 pg/ml; P = 0.014).

In the control group, the serum sRAGE level of the subjects with the rs2070600 AG genotype was significantly lower (10.96 ± 39.47 pg/ml) than that of the subjects with the GG genotype (90.56 ± 183.54 pg/ml) (P = 0.024). A further analysis revealed that the serum sRAGE level was significantly higher in individuals with rs184003 homozygous TT genotypes (124.63 ± 200.63 pg/ml) and rs184003 heterozygous GT genotypes (119.50 ± 254.34 pg/ml) than homozygous GG genotypes (32.46 ± 77.92 pg/ml; P = 0.044 and P = 0.016, respectively). These results demonstrated that circulating sRAGE levels were largely determined by *RAGE* genetic defects.

## Discussion

This study examined the influence of four well-characterized polymorphisms of the *RAGE* gene on gastric cancer risk and investigated whether these polymorphisms were correlated with serum sRAGE levels in a southwestern Han Chinese population. There were four main findings in this study. First, they suggested that the rs2070600 variant AG genotype might play a predominant role in the development of gastric cancer (OR 1.62, 95% CI 1.03–2.58). In contrast, the rs184003 GT genotype represented a significantly reduced risk for gastric cancer (OR 0.62, 95% CI 0.39–0.99). Second, the rs2070600 AG genotype enhanced the risk of gastric cancer among nonsmokers (OR 1.71, 95% CI 1.01–2.91), nondrinkers (OR 1.75, 95% CI 1.03–2.97), and patients were at tumor stage III (OR 2.00, 95% CI 1.13–3.56). Third, the average sRAGE levels in the gastric cancer patients were significantly decreased compared with those of the healthy controls. Fourth, the rs2070600 and rs184003 polymorphisms appeared to affect the serum levels of sRAGE.

In the present study, the ability to produce sRAGE was decreased in the subjects carrying the rs2070600 AG genotype, whereas it was increased in those carrying the rs184003 T variant allele. These findings suggest that the *RAGE* rs2070600 and rs184003 genetic polymorphisms could potentially be used as genetic markers for gastric cancer.

RAGE acts as a link between inflammatory pathways and pathways promoting tumor progression and metastasis [4]. Previous research revealed that RAGE was one of the key factors accelerating tumorigenesis and metastasis in various diseases [5]. In recent years, the relationship between *RAGE* gene polymorphisms and tumorigenesis has attracted significant attention [5], and increasing evidence points to the role of genetic variants in the *RAGE* gene altering the expression and function of sRAGE, thus affecting disease development [25–29]. In addition, several epidemiological studies have investigated the association between the *RAGE* gene polymorphism and risk of various cancers. For example, in 2014, Qian et al. [16] found that the *RAGE* rs2070600 AG and AA genotypes significantly increased the risk of colorectal cancer, with ORs of 2.037 (95% CI 1.21–3.44) and 1.207 (95% CI 0.94–11.65), respectively. Zhang [17] found an association between the rs2070600 variant genotypes and a significantly decreased risk of epithelial ovarian cancer (OR 2.65, 95% CI 1.54–4.58, P = 0.0004). In addition, Wang et al. [18] reported that subjects carrying the rs2070600 AG genotype had a significantly higher risk of developing nonsmall cell lung cancer (OR 1.72, 95% CI 1.21–2.44; P = 0.002). In the present study, the rs2070600 AG genotype was significantly associated with the risk of gastric cancer, which was in accordance with the results in the literature.

In a case–control study, Pan et al. [14] demonstrated an association between the rs184003 T allele and breast cancer, with an OR of 1.62 (95% CI 1.26–2.08; P < 0.001). In the present study, the rs184003 G allele was associated with a significantly reduced risk of gastric cancer, and the wild-type T allele denoted a significantly increased risk of the disease. These findings are in accordance with those of Pan et al. [14].

Su et al. [13] provided evidence that the rs1800625 CT and CC genotypes were associated with the risk and progression of hepatocellular carcinoma (adjusted OR 2.568, 95% CI 1.418–4.653; and adjusted OR 2.808, 95% CI 1.581–4.985, respectively). Su et al. [15] found that individuals carrying the polymorphic allele of rs1800625 were more susceptible to oral cancer (adjusted OR 2.053, 95% CI 1.269–3.345). However, the present study did not find any significant association between the rs1800625 polymorphism and gastric cancer risk.

In a meta-analysis of 27 studies performed in 2015, the rs2070600 (82G/S) polymorphism was associated with a significantly increased risk of cancer (A vs. G: OR 1.321, 95% CI 1.164–1.499; AA vs. GG: OR 1.823, 95% CI 1.541–2.157; AG vs. GG: OR 1.399, 95% CI 1.120–1.746; GA + AA vs. GG: OR 1.470, 95% CI 1.187–1.821; AA vs. GG + AG: OR 1.416, 95% CI 1.158–1.732) [30]. In the same study, the rs1800624 (−374T/A) polymorphism was associated with a reduced risk of cancer (AA vs. TT: OR 0.818, 95% CI 0.686–0.976; A vs. T: OR 0.908, 95% CI 0.840–0.981). Furthermore, the subgroup analysis revealed a significantly elevated risk of lung cancer with the rs2070600 (82G/S) polymorphism in an Asian population. The subgroup analysis also showed that the rs1800624 (−374T/A) polymorphism seemed to be associated with a reduced risk in a Caucasian population and in patients with lung cancer and breast cancer [30].

Only one previous study performed in 2008 investigated the association between *RAGE* polymorphisms and gastric cancer risk [22]. In that study, Gu et al. included 283 gastric cancer patients and 283 age- and sex-matched controls and reported that subjects with the rs2070600 variant genotypes (82Gly/Ser and 82Ser/Ser) had a significantly higher risk of gastric cancer (adjusted OR 1.47, 95% CI 1.05–2.06). Moreover, the elevated gastric cancer risk was especially evident among younger individuals (aged ≤58 years), nonsmokers, and rural-dwelling subjects. However, Gu et al. evaluated only one SNP (rs2070600, 82Gly/Ser) of the RAGE gene in a northeastern Chinese population. The present study investigated the association between four common SNPs rs1800624 (−374T>A), rs1800625 (−429T>C), rs184003 (1704G>T), and rs2070600 (Gly82Ser) of the *RAGE* gene and gastric cancer risk in a southwest Han Chinese population. The findings pointed to a significant association between the rs2070600 polymorphisms and risk of gastric cancer development, especially in nonsmokers and nondrinkers, which was similar to the results of Gu et al. [22]. In addition, the results of the present study indicated that the rs184003 variant genotypes were significantly associated with the risk of gastric cancer. As the present study included a larger number of SNPs, more sufficient results were obtained in the present study than in the previous study.

Some studies revealed that the expression of *RAGE* increased with tumor progression, depth of tumor invasion, and presence of metastasis in lymph nodes in patients with prostate cancer [6], gallbladder cancer [7], colorectal cancer [31], pancreatic cancer [8], and most gastric cancer cell lines [5]. On the other hand, in cells of human nonsmall cell lung cancer, the expression of *RAGE* was decreased [9], and induced expression of *RAGE* decreased the rate of growth of tumor cells [32] and limited the proliferation of lung fibroblasts [33].

In a prospective case-cohort study of 29,133 Finnish male smokers, higher prediagnostic levels of serum sRAGE were associated with a lower risk of colorectal cancer [10]. Other findings also suggested that sRAGE was inversely associated with the risk of pancreatic cancer among Finnish male smokers [34]. Wagner et al. [35] demonstrated that sRAGE and variants at its genetic locus were prognostic markers for survival in melanoma patients with a high risk of progression. Wang et al. [36] observed that sRAGE levels were downregulated in serum and that the expression of *RAGE* was decreased in lung cancer tissue. In the latest meta-analysis of 15 studies by Huang et al. in 2016 [19], carriers of the rs2070600 (Gly82Ser) AA (82Ser/82Ser) genotype had significantly reduced circulating sRAGE concentrations compared with the GG (82Gly/82Gly) genotype. In Huang et al.’s research, Mendelian randomization analysis demonstrated that a reduction of 100, 200 and 300 pg/ml in circulating sRAGE concentrations was associated with a 1.11-fold (95% CI 1.06, 1.25), 1.24-fold (95% CI 1.11, 1.57), and 1.38-fold (95% CI 1.18, 1.96) increased risk of developing cancer, respectively [29]. In the present study, the average sRAGE levels in the gastric cancer patients were significantly decreased compared to those of the healthy controls. Subjects carrying the rs2070600 (Gly82Ser) variant genotype had a decreased ability to produce sRAGE. The results of the present study are in accordance with the idea mentioned above.

In the current study, the rs2070600 AG genotype was significantly associated with an increased risk of gastric cancer among nonsmokers (adjusted OR 1.71, 95% CI 1.01–2.91), nondrinkers (adjusted OR 1.75, 95% CI 1.03–2.97) and gastric cancer patients with stage III disease (OR 2.00 95% CI 1.13–3.56). The evidence for different effects of gender, smoking, drinking status, and cancer stage on gastric cancer risk was suggestive but not conclusive. The limited number of subjects in the study may explain this finding. After stratifying the population by gender, smoking, drinking status, and cancer stage, the sample size in each subgroup was small. Thus, the results lack statistical power and robustness. The mechanisms underlying different effects of various variables (gender, smoking, drinking status, and cancer stage) on gastric cancer risk remain unknown. The current findings must be interpreted in light of several potential limitations. The study was limited to eligible participations in Guangxi, which might not be representative of the entire Chinese population. Another limitation was the measurement of circulating sRAGE levels, which were measured only once, making it impossible to reflect on the long-term effect of sRAGE levels on the development of gastric cancer. A third limitation was that this research was based on data from individual participants and only four SNPs of the *RAGE* gene, which restricted interpretations about gene-to-gene or gene-to-environment interactions. These limitations restrict the interpretation and extrapolation of the current findings.

## Conclusions

In conclusion, the current findings demonstrated that *RAGE* rs2070600 variant genotypes might play a predominant role in the development of gastric cancer. In contrast, the rs184003 variant genotypes represented a significantly reduced risk for gastric cancer. sRAGE levels were inversely associated with gastric cancer risk. Genetically lowered concentrations of circulating sRAGE might confer an increased risk of gastric cancer.

## Additional files

**Additional file 1.** The genotype distributions of RAGE polymorphisms estimated by gender.

**Additional file 2.** The genotype distributions of RAGE polymorphisms estimated by smoking status.

**Additional file 3.** The genotype distributions of RAGE polymorphisms estimated by drinking status.

**Additional file 4.** Stratified analyses for RAGE genotypes polymorphisms in cases and controls by cancer stage.

## Abbreviations

AGE: advanced glycation end products
BMI: body mass index
CI: confidence interval
OR: odds ratio
PCR-RFLP: polymerase chain reaction-restriction fragment length polymorphism
RAGE: receptor for advanced glycation end products
SNP: single-nucleotide polymorphism
