# Supplementary material for: Effect of RAGE gene polymorphisms and circulating sRAGE levels on susceptibility to gastric cancer: a case–control study
Source: Cancer Cell Int. 2017 Feb 6;17:19. doi: 10.1186/s12935-017-0391-0 (PMC5294806; doi:10.1186/s12935-017-0391-0)
Supplement: Supplementary file 1 — Additional file 1. The genotype distributions of RAGE polymorphisms estimated by gender. [file 12935_2017_391_MOESM1_ESM.docx]

**Online Resource 1.** The genotype distributions of RAGE polymorphisms estimated by gender

| Model | Males | | | |  | Females | | | |
| --- | --- | --- | --- | --- | --- | --- | --- | --- | --- |
|  | Control (N=97) | Cases (N= 131) | OR (95% CI) ^*^ | *P* |  | Control (N=110) | Cases (N= 69) | OR (95% CI) ^*^ | *P* |
| **rs2070600** |  |  |  |  |  |  |  |  |  |
| GG | 64 0.660) | 74 (0.565) | 1.00^ref^ |  |  | 72 (0.655) | 39 (0.565) | 1.00^ref^ |  |
| AG | 27 (0.278) | 44 (0.336) | 1.71 (0.91-3.19) | 0.095 |  | 31 (0.282) | 28 (0.406) | 1.40 (0.64-3.03) | 0.398 |
| AA | 6 (0.062) | 13 (0.099) | 1.72 (0.58-5.10) | 0.325 |  | 7 (0.064) | 2 (0.029) | 0.58 (0.84-4.02) | 0.581 |
| A allele | 39 (0.201) | 70 (0.267) | 1.00^ref^ |  |  | 45 (0.205) | 32 (0.232) | 1.00^ref^ |  |
| G allele | 155 (0.799) | 192 (0.733) | 1.53 (0.96-2.45) | 0.076 |  | 175 (0.795) | 106 (0.768) | 1.19 (0.64-2.23) | 0.584 |
| AG+AA vs. GG |  |  | 1.71 (0.96-3.05) | 0.071 |  |  |  | 1.28 (0.61-2.71) | 0.517 |
| AA vs. AG+GG |  |  | 1.45 (0.50-4.18) | 0.496 |  |  |  | 0.91 (0.12-7.12) | 0.926 |
| **rs184003** |  |  |  |  |  |  |  |  |  |
| GG | 63 (0.649) | 95 (0.725) | 1.00^ref^ |  |  | 75 (0.682) | 53 (0.768) | 1.00^ref^ |  |
| GT | 33 (0.340) | 34 (0.260) | 0.68 (0.37-1.24) | 0.209 |  | 31 (0.282) | 14 (0.203) | 0.54 (0.22-1.31) | 0.172 |
| TT | 1 (0.010) | 2 (0.015) | 1.31 (0.11-15.85) | 0.830 |  | 4 (0.036) | 2 (0.029) | 0.54 (0.08-3.88) | 0.539 |
| G allele | 159 (0.820) | 224 (0.855) | 1.00^ref^ |  |  | 181 (0.823) | 120 (0.870) | 1.00^ref^ |  |
| T allele | 35 (0.180) | 38 (0.145) | 0.77 (0.45-1.30) | 0.326 |  | 39 (0.177) | 18 (0.130) | 0.56 (0.27-1.17) | 0.120 |
| GT+TT vs. GG |  |  | 0.70 (0.39-1.27) | 0.237 |  |  |  | 0.54 (0.23-1.25) | 0.148 |
| TT vs. GT+GG |  |  | 1.47 (0.12-17.66) | 0.761 |  |  |  | 0.59 (0.08-4.22) | 0.599 |
| **rs1800624** |  |  |  |  |  |  |  |  |  |
| TT | 75 (0.773) | 99 (0.756) | 1.00^ref^ |  |  | 91 (0.827) | 51 (0.739) | 1.00^ref^ |  |
| AT | 17 (0.175) | 25 (0.191) | 1.01 (0.49-2.10) | 0.976 |  | 18 (0.164) | 17 (0.246) | 1.83 (0.71-4.69) | 0.209 |
| AA | 5 (0.052) | 7 (0.053) | 0.78 (0.21-2.87) | 0.709 |  | 1 (0.009) | 1 (0.014) | 4.55 (0.08-265.64) | 0.466 |
| T allele | 167 (0.861) | 223 (0.851) | 1.00^ref^ |  |  | 200 (0.909) | 119 (0.862) | 1.00^ref^ |  |
| A allele | 27 (0.139) | 39 (0.149) | 0.92 (0.53-1.63) | 0.785 |  | 20 (0.091) | 19 (0.138) | 1.83 (0.79-4.25) | 0.160 |
| AA+AT vs. TT |  |  | 0.96 (0.49-1.86) | 0.898 |  |  |  | 1.97 (0.79-4.89) | 0.146 |
| AA vs. AT+TT |  |  | 0.78 (0.21-2.85) | 0.705 |  |  |  | 4.25 (0.07-256.64) | 0.489 |
| **rs1800625** |  |  |  |  |  |  |  |  |  |
| CC | 1 (0.010) | 3 (0.023) | 1.00^ref^ |  |  | 0 (0.00) | 0 (0.00) | 1.00^ref^ |  |
| CT | 10 (0.103) | 10 (0.076) | 0.43 (0.03-5.76) | 0.520 |  | 12 (0.109) | 3 (0.043) | NA |  |
| TT | 86 (0.887) | 118 (0.901) | 0.62 (0.05-7.22) | 0.701 |  | 98 (0.891) | 66 (0.957) | NA |  |
| C allele | 12 (0.062) | 16 (0.061) | 1.00^ref^ |  |  | 12 (0.055) | 3 (0.022) | 1.00^ref^ |  |
| T allele | 182 (0.938) | 246 (0.939) | 1.16 (0.51-2.63) | 0.721 |  | 208 (0.945) | 135 (0.978) | 2.97 (0.66-13.40) | 0.157 |
| TT+CT vs. CC |  |  | 0.60 (0.051-6.93) | 0.680 |  |  |  | NA |  |
| TT vs. CT+CC |  |  | 1.29 (0.53-3.17) | 0.576 |  |  |  | 3.14 (0.67-14.77) | 0.147 |

^*^Adjusted for age, BMI, family history of cancer, ethnicity, smoking and drinking status.
