# Supplementary material for: Effect of RAGE gene polymorphisms and circulating sRAGE levels on susceptibility to gastric cancer: a case–control study
Source: Cancer Cell Int. 2017 Feb 6;17:19. doi: 10.1186/s12935-017-0391-0 (PMC5294806; doi:10.1186/s12935-017-0391-0)
Supplement: Supplementary file 2 — Additional file 2. The genotype distributions of RAGE polymorphisms estimated by smoking status. [file 12935_2017_391_MOESM2_ESM.docx]

**Online Resource 2.** The genotype distributions of RAGE polymorphisms estimated by smoking status

| Model | Smokers | | | |  | Never smoker | | | |
| --- | --- | --- | --- | --- | --- | --- | --- | --- | --- |
|  | Control (N=64) | Cases (N= 59) | Adjusted OR (95% CI) ^*^ | *P* |  | Control (N=143) | Cases (N= 141) | Adjusted OR (95% CI) ^*^ | *P* |
| **rs2070600** |  |  |  |  |  |  |  |  |  |
| GG | 42 (0.656) | 34 (0.576) | 1.00^ref^ |  |  | 94 (0.657) | 79 (0.560) | 1.00^ref^ |  |
| AG | 15 (0.234) | 16 (0.271) | 0.91 (0.31-2.68) | 0.867 |  | 43 (0.301) | 56 (0.397) | 1.71 (1.01-2.91) | **0.043** |
| AA | 7 (0.109) | 9 (0.153) | 1.70 (0.39-7.35) | 0.476 |  | 6 (0.042) | 6 (0.043) | 0.91 (0.27-3.03) | 0.881 |
| A allele | 29 (0.227) | 34 (0.288) | 1.00^ref^ |  |  | 55 (0.192) | 68 (0.241) | 1.00^ref^ |  |
| G allele | 99 (0.773) | 84 (0.712) | 1. 26-2.67 (0.5) | 0.553 |  | 231 (0.808) | 214 (0.759) | 1.33 (0.86-2.03) | 0.197 |
| AG+AA vs. GG |  |  | 1.11 (0.43-2.90) | 0.825 |  |  |  | 1.53 (0.92-2.56) | 0.104 |
| AA vs. AG+GG |  |  | 1.76 (0.42-7.27) | 0.440 |  |  |  | 0.87 (0.25-3.07) | 0.834 |
| **rs184003** |  |  |  |  |  |  |  |  |  |
| GG | 42 (0.656) | 43 (0.729) | 1.00^ref^ |  |  | 4 (0.028) | 105 (0.745) | 1.00^ref^ |  |
| GT | 21 (0.328) | 15 (0.254) | 0.56 (0.20-1.59) | 0.277 |  | 43 (0.301) | 33 (0.234) | 0.64 (0.36-1.11) | 0.114 |
| TT | 1 (0.016) | 1 (0.017) | 3.06 (0.03-348.82) | 0.644 |  | 96 (0.671) | 3 (0.021) | 0.74(0.15-3.67) | 0.715 |
| G allele | 105 (0.820) | 101 (0.856) | 1.00^ref^ |  |  | 235 (0.822) | 243 (0.862) | 1.00^ref^ |  |
| T allele | 23 (0.180) | 17 (0.144) | 0.71 (0.29-1.72) | 0.448 |  | 51 (0.178) | 39 (0.138) | 0.68 (0.42-1.10) | 0.116 |
| GT+TT vs. GG |  |  | 0.60 (0.22-1.66) | 0.324 |  |  |  | 0.63 (0.36-1.09) | 0.101 |
| TT vs. GT+GG |  |  | 3.71 (0.03-476.66) | 0.597 |  |  |  | 0.70 (0.14-3.44) | 0.662 |
| **rs1800624** |  |  |  |  |  |  |  |  |  |
| TT | 50 (0.781) | 40 (0.678) | 1.00^ref^ |  |  | 116 (0.811) | 110 (0.780) | 1.00^ref^ |  |
| AT | 11 (0.172) | 14 (0.237) | 1.13 (0.36-3.60) | 0.833 |  | 24 (0.168) | 28 (0.199) | 1.31 (0.70-2.46) | 0.397 |
| AA | 3 (0.047) | 5 (0.085) | 0.68 (0.10-4.75) | 0.698 |  | 3 (0.021) | 3 (0.021) | 1.10(0.20-5.79) | 0.935 |
| T allele | 111 (0.867) | 94 (0.797) | 1.00^ref^ |  |  | 256 (0.895) | 248 (0.879) | 1.00^ref^ |  |
| A allele | 17 (0.133) | 24 (0.203) | 0.93 (0.39-2.21) | 0.866 |  | 30 (0.105) | 34 (0.121) | 1.19 (0.69-2.05) | 0.543 |
| AA+AT vs. TT |  |  | 1.00 (0.36-2.85) | 0.988 |  |  |  | 1.22 (0.67-2.25) | 0.515 |
| AA vs. AT+TT |  |  | 0.67 (0.00-4.60) | 0.680 |  |  |  | 1.07 (0.20-5.87) | 0.938 |
| **rs1800625** |  |  |  |  |  |  |  |  |  |
| CC | 0 (0.000) | 2 (0.034) | 1.00^ref^ |  |  | 1 (0.007) | 1 (0.007) | 1.00^ref^ |  |
| CT | 7 (0.109) | 5 (0.085) | NA |  |  | 15 (0.105) | 8 (0.057) | 0.35 (0.02-7.15) | 0.494 |
| TT | 57 (0.891) | 52 (0.881) | NA |  |  | 127 (0.888) | 132 (0.936) | 0.66 (0.04-11.97) | 0.777 |
| C allele | 7 (0.055) | 9 (0.076) | 1.00^ref^ |  |  | 17 (0.059) | 10 (0.035) | 1.00^ref^ |  |
| T allele | 121 (0.945) | 109 (0.924) | 0.96 (0.25-3.77) | 0.958 |  | 269 (0.941) | 272 (0.965) | 1.43 (0.62-3.32) | 0.400 |
| TT+CT vs. CC |  |  | NA |  |  |  |  | 0.78 (0.04-14.18) | 0.864 |
| TT vs. CT+CC |  |  | 1.13 (0.26-4.92) | 0.867 |  |  |  | 1.55 (0.63-3.81)) | 0.342 |

^*^Adjusted for gender, age, BMI, family history of cancer, ethnicity, and drinking status.
