# Supplementary material for: Effect of RAGE gene polymorphisms and circulating sRAGE levels on susceptibility to gastric cancer: a case–control study
Source: Cancer Cell Int. 2017 Feb 6;17:19. doi: 10.1186/s12935-017-0391-0 (PMC5294806; doi:10.1186/s12935-017-0391-0)
Supplement: Supplementary file 3 — Additional file 3. The genotype distributions of RAGE polymorphisms estimated by drinking status. [file 12935_2017_391_MOESM3_ESM.docx]

**Online Resource 3.** The genotype distributions of RAGE polymorphisms estimated by drinking status

| Model | Drinkers | | | |  | Never drinkers | | | |
| --- | --- | --- | --- | --- | --- | --- | --- | --- | --- |
|  | Control (N=58) | Cases (N= 52) | Adjusted OR (95% CI) ^*^ | *P* |  | Control (N=149) | Cases (N= 148) | Adjusted OR (95% CI) ^*^ | *P* |
| **rs2070600** |  |  |  |  |  |  |  |  |  |
| GG | 39 (0.672) | 32 (0.615) | 1.00^ref^ |  |  | 97 (0.651) | 81 (0.547) | 1.00^ref^ |  |
| AG | 16 (0.276) | 14 (0.269) | 1.17 (0.39-3.52) | 0.774 |  | 42 (0.282) | 58 (0.392) | 1.75 (1.03-2.97) | **0.037** |
| AA | 3 (0.052) | 6 (0.115) | 1.99 (0.30-13.45) | 0.478 |  | 10 (0.067) | 9 (0.061) | 1.23 (0.43-3.52) | 0.699 |
| A allele | 22 (0.190) | 26 (0.250) | 1.00^ref^ |  |  | 62 (0.208) | 76 (0.257) | 1.00^ref^ |  |
| G allele | 94 (0.810) | 78 (0.750) | 1.31 (0.63-2.74) | 0.465 |  | 236 (0.792) | 220 (0.743) | 1.43 (0.95-2.15) | 0.087 |
| AG+AA vs. GG |  |  | 1.32 (0.48-3.62) | 0.590 |  |  |  | 1.66 (1.01-2.74) | **0.047** |
| AA vs. AG+GG |  |  | 1.91 (0.29-12.56) | 0.501 |  |  |  | 1.06 (0.38-2.96) | 0.916 |
| **rs184003** |  |  |  |  |  |  |  |  |  |
| GG | 39 (0.672) | 38 (0.731) | 1.00^ref^ |  |  | 99 (0.664) | 110 (0.743) | 1.00^ref^ |  |
| GT | 19 (0.328) | 13 (0.250) | 0.71 (0.24-2.05) | 0.522 |  | 45 (0.302) | 35 (0.236) | 0.63 (0.36-1.09) | 0.100 |
| TT | 0 (0.000) | 1 (0.019) | NA |  |  | 5 (0.034) | 3 (0.020) | 0.60 (0.13-2.91) | 0.530 |
| G allele | 97 (0.836) | 89 (0.856) | 1.00^ref^ |  |  | 243 (0.815) | 255 (0.861) | 1.00^ref^ |  |
| T allele | 19 (0.164) | 15 (0.144) | 0.82 (0.35-1.92) | 0.639 |  | 55 (0.185) | 41 (0.139) | 0.68 (0.42-1.08) | 0.102 |
| GT+TT vs. GG |  |  | 0.77 (0.27-2.18) | 0.618 |  |  |  | 0.63 (0.37-1.07) | 0.087 |
| TT vs. GT+GG |  |  | NA |  |  |  |  | 0.64 (0.14-3.01) | 0.573 |
| **rs1800624** |  |  |  |  |  |  |  |  |  |
| TT | 43 (0.741) | 40 (0.769) | 1.00^ref^ |  |  | 123 (0.826) | 110 (0.743) | 1.00^ref^ |  |
| AT | 14 (0.241) | 9 (0.173) | 0.55 (0.16-1.85) | 0.333 |  | 21 (0.141) | 33 (0.223) | 1.85 (0.98-3.52) | 0.059 |
| AA | 1 (0.017) | 3 (0.058) | 0.99 (0.08-11.97) | 0.996 |  | 5 (0.034) | 5 (0.034) | 1.04 (0.26-4.15) | 0.959 |
| T allele | 100 (0.862) | 89 (0.856) | 1.00^ref^ |  |  | 267 (0.896) | 253 (0.855) | 1.00^ref^ |  |
| A allele | 16 (0.138) | 15 (0.144) | 0.70 (0.29-1.67) | 0.419 |  | 31 (0.104) | 43 (0.145) | 1.51 (0.90-2.55) | 0.118 |
| AA+AT vs. TT |  |  | 0.61 (0.20-1.88) | 0.387 |  |  |  | 1.70 (0.94-3.08) | 0.082 |
| AA vs. AT+TT |  |  | 1.15 (0.10-13.45) | 0.912 |  |  |  | 1.02 (0.26-3.98) | 0.980 |
| **rs1800625** |  |  |  |  |  |  |  |  |  |
| CC | 0 (0.000) | 2 (0.038) | 1.00^ref^ |  |  | 1 (0.007) | 1 (0.007) | 1.00^ref^ |  |
| CT | 7 (0.121) | 4 (0.077) | NA |  |  | 15 (0.101) | 9 (0.061) | 0.52 (0.02-11.14) | 0.677 |
| TT | 51 (0.879) | 46 (0.885) | NA |  |  | 133 (0.893) | 138 (0.932) | 0.81 (0.04-15.16) | 0.886 |
| C allele | 7 (0.060) | 8 (0.077) | 1.00^ref^ |  |  | 17 (0.057) | 11 (0.037) | 1.00^ref^ |  |
| T allele | 109 (0.940) | 96 (0.923) | 0.99 (0.30-3.20) | 0.982 |  | 281 (0.943) | 285 (0.963) | 1.37 (0.61-3.08) | 0.448 |
| TT+CT vs. CC |  |  | NA |  |  |  |  | 0.79 (0.04-14.86) | 0.877 |
| TT vs. CT+CC |  |  | 1.68 (0.43-6.53) | 0.453 |  |  |  | 1.47 (0.62-3.49) | 0.389 |

^*^Adjusted for age, BMI, family history of cancer, ethnicity, and smoking status.
