# Supplementary material for: Effect of RAGE gene polymorphisms and circulating sRAGE levels on susceptibility to gastric cancer: a case–control study
Source: Cancer Cell Int. 2017 Feb 6;17:19. doi: 10.1186/s12935-017-0391-0 (PMC5294806; doi:10.1186/s12935-017-0391-0)
Supplement: Supplementary file 4 — Additional file 4. Stratified analyses for RAGE genotypes polymorphisms in cases and controls by cancer stage. [file 12935_2017_391_MOESM4_ESM.docx]

**Online Resource 4.** Stratified analyses for RAGE genotypes polymorphisms in cases and controls by cancer stage

| Model | Controls (N=207) | State I cases | | | State II cases | | | State III cases | | | State IV cases | | |
| --- | --- | --- | --- | --- | --- | --- | --- | --- | --- | --- | --- | --- | --- |
|  |  | N =26 | OR (95% CI) * | P | N=35 | OR (95% CI) * | P | N=85 | OR (95% CI) * | P | N=54 | OR (95% CI) * | P |
| rs2070600 |  |  |  |  |  |  |  |  |  |  |  |  |  |
| GG | 136 | 15 | 1.00^ref^ |  | 20 | 1.00^ref^ |  | 46 | 1.00^ref^ |  | 32 | 1.00^ref^ |  |
| AG | 58 | 10 | 1.58 (0.64-3.89) | 0.322 | 12 | 1.74 (0.74-4.09) | 0.208 | 33 | 2.00 (1.13-3.56) | **0.018** | 17 | 1.47 (0.72-3.02) | 0.296 |
| AA | 13 | 1 | 0.74 (0.09-6.37) | 0.786 | 3 | 1.76 (0.42-7.50) | 0.442 | 6 | 1.26 (0.42-3.74) | 0.683 | 5 | 1.83 (0.54-6.23) | 0.332 |
| rs184003 |  |  |  |  |  |  |  |  |  |  |  |  |  |
| GG | 138 | 16 | 1.00^ref^ |  | 27 | 1.00^ref^ |  | 68 | 1.00^ref^ |  | 37 | 1.00^ref^ |  |
| GT | 64 | 9 | 1.11 (0.45-2.75) | 0.817 | 7 | 0.54 (0.21-1.39) | 0.204 | 17 | 0.54 (0.27-1.02) | 0.057 | 15 | 0.80 (0.39-1.63) | 0.535 |
| TT | 5 | 1 | 2.92 (0.29-29.78) | 0.365 | 1 | 1.64 (1.16-17.18) | 0.678 | 0 | NA |  | 2 | 1.61 (0.26-9.97) | 0.608 |
| rs1800624 |  |  |  |  |  |  |  |  |  |  |  |  |  |
| TT | 166 | 22 | 1.00^ref^ |  | 21 | 1.00^ref^ |  | 66 | 1.00^ref^ |  | 41 | 1.00^ref^ |  |
| AT | 35 | 4 | 0.83 (0.26-2.65) | 0.746 | 9 | 2.03 (0.81-5.11) | 0.132 | 16 | 1.16 (0.59-2.31) | 0.669 | 14 | 1.84 (0.85-4.02) | 0.123 |
| AA | 6 | 0 | NA |  | 5 | 3.69 (0.83-16.51) | 0.088 | 3 | 0.99 (0.22-4.48) | 0.989 | 0 | NA |  |
| rs1800625 |  |  |  |  |  |  |  |  |  |  |  |  |  |
| CC | 1 | 1 | 1.00^ref^ |  | 0 | 1.00^ref^ |  | 2 | 1.00^ref^ |  | 0 | 1.00^ref^ |  |
| CT | 22 | 1 | 0.67 (0.001-3.00) | 0.163 | 1 | NA |  | 5 | 0.14 (0.01-2.07) | 0.154 | 6 | NA |  |
| TT | 184 | 22 | 0.18 (0.007-4.60) | 0.296 | 34 | NA |  | 78 | 0.27 (0.02-3.27) | 0.302 | 48 | NA |  |

^*^Adjusted for age, gender, BMI, family history of cancer, ethnicity, drinking and smoking status.

NA, not application

Boldfaced values indicate a significant difference.
